# Chemotherapy-induced cardiotoxicity: a new perspective on the role of Digoxin, ATG7 activators, Resveratrol, and herbal drugs

**DOI:** 10.25122/jml-2022-0322

**Published:** 2023-04

**Authors:** Hany Akeel Al-hussaniy, Ali Hikmat Alburghaif, Zahraa alkhafaje, Mohammed Abdul-Hassan Jabarah AL-Zobaidy, Hayder Mutair Alkuraishy, Gomaa Mostafa-Hedeab, Faizul Azam, Ali Mahmoud Al-Samydai, Zahraa Salam Al-tameemi, Meena Akeel Naji

**Affiliations:** 1 Department of Pharmacy, Bilad Alrafidain University College, Diyala, Iraq; 2 Dr. Hany Akeel Institute, Iraqi Medical Research Center, Baghdad, Iraq; 3 Department of Pharmacy, Ashur University College, Baghdad, Iraq; 4 Department of Pharmacy, Alfarahidi University College, Baghdad, Iraq; 5 Department of Clinical Pharmacology, College of Medicine, Almustansria University, Baghdad, Iraq; 6 Pharmacology Department & Health Research Unit, Medical College, Jouf University, Jouf, Saudi Arabia; 7 Pharmacology Department, Faculty of Medicine, Beni-Suef University, Beni-Suef, Egypt; 8 Department of Pharmaceutical Chemistry and Pharmacognosy, Unaizah College of Pharmacy, Qassim University, Uniazah, Saudi Arabia; 9 Pharmacological and Diagnostic Research Centre, Faculty of Pharmacy, Al-Ahliyya Amman University, Amman, Jordan

**Keywords:** cardiotoxicity, anthracyclines, neoadjuvant therapy, free radicals

## Abstract

Cancer is a major public health problem, and chemotherapy plays a significant role in the management of neoplastic diseases. However, chemotherapy-induced cardiotoxicity is a serious side effect secondary to cardiac damage caused by antineoplastic's direct and indirect toxicity. Currently, there are no reliable and approved methods for preventing or treating chemotherapy-induced cardiotoxicity. Understanding the mechanisms of chemotherapy-induced cardiotoxicity may be vital to improving survival. The independent risk factors for developing cardiotoxicity must be considered to prevent myocardial damage without decreasing the therapeutic efficacy of cancer treatment. This systematic review aimed to identify and analyze the evidence on chemotherapy-induced cardiotoxicity, associated risk factors, and methods to decrease or prevent it. We conducted a comprehensive search on PubMed, Google Scholar, and Directory of Open Access Journals (DOAJ) using the following keywords: “doxorubicin cardiotoxicity”, “anthracycline cardiotoxicity”, “chemotherapy”, “digoxin decrease cardiotoxicity”, “ATG7 activators”, retrieving 59 articles fulfilling the inclusion criteria. Therapeutic schemes can be changed by choosing prolonged infusion application over boluses. In addition, some agents like Dexrazoxane can reduce chemotherapy-induced cardiotoxicity in high-risk groups. Recent research found that Digoxin, ATG7 activators, Resveratrol, and other medical substances or herbal compounds have a comparable effect on Dexrazoxane in anthracycline-induced cardiotoxicity.

## INTRODUCTION

Cancer is a significant global public health issue, causing over 10 million deaths in 2020, and is projected to surpass cardiovascular disease as the leading cause of death by 2025 to 2030 [1]. According to the World Health Organization (WHO), in 2019, cancer was the first leading cause of death in 57 countries, including the US, Canada, and Europe, and the second cause of death after cardiovascular diseases in 55 other countries [2,3]. Although the age-adjusted incidence rate has decreased by about 31%, the number of cancer cases continues to grow. This is associated with an older population and increased survival due to scientific advances in the early detection of cancer [4,5]. However, despite the improved survival, cancer therapy has revealed significant cardiovascular toxicities that were previously overlooked.

Chemotherapy and radiotherapy are two of the mainstays of treatment for several types of cancer. These treatments have allowed for an increased number of patients to survive. However, their mechanism, doses, and frequency of use to achieve remission can generate side effects in patients, with cardiotoxicity as one of the most concerning [5,6]. These side effects can manifest as symptoms of heart failure and myocardial damage from direct and indirect toxicity of antineoplastic therapy [7,8]. Because of this, cardiac function is considered a dose-limiting variable during cancer therapy, contributing to the exposed population's morbidity and mortality [9].

Cardiotoxicity encompasses various pathological manifestations at the cardiovascular level caused by oncological treatment, with heart failure being the most frequent complication linked with a 3.5-fold increase in mortality with anthracycline therapy [10]. A study on 22,643 adult survivors of childhood cancer showed an increasing prevalence of heart conditions over time, from less than 3% at the age of 20 to 8.8–9.0% in those 35 years of age [10].

Cardiovascular evaluation of chemotherapy patients, risk assessment, mitigation of cardiac harm, and monitoring of heart function before, during, and after chemotherapy should be performed. Additionally, to ensure a comprehensive approach to patient care and promote positive outcomes, multidisciplinary efforts are needed to further develop existing pharmaceuticals and devise new strategies for preventing and treating cardiotoxicity. Cardio-oncology has become a crucial field in providing comprehensive care for chemotherapy patients [11].

The study aimed to systematically review the literature on chemotherapy-induced cardiotoxicity and compare as well as identify the chemotherapies that cause cardiotoxicity along with their mechanisms and methods for decreasing or preventing it.

## Material and Methods

**Figure 1:**
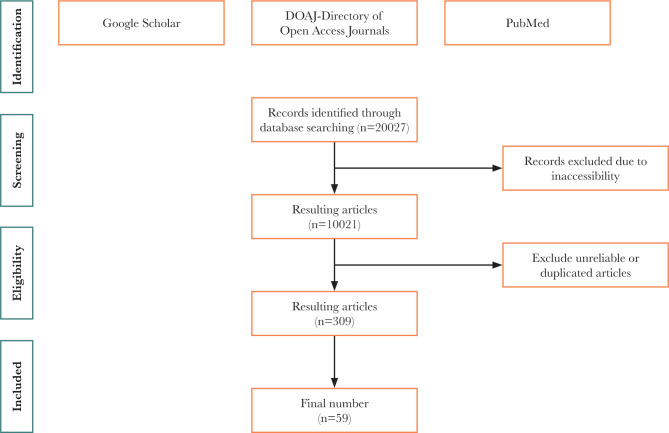
Selection criteria for inclusion of articles.

We searched for relevant articles in PubMed, Google Scholar, and Directory of Open Access Journals (DOAJ) using keywords such as "doxorubicin cardiotoxicity", "anthracycline cardiotoxicity", "chemotherapy", "digoxin decrease cardiotoxicity", and "ATG7 activators". To ensure the quality and reliability of the articles, we excluded articles that were inaccessible, outdated, unreliable, or not directly related to our study's focus (e.g., articles on gene polymorphism or other side effects of chemotherapy). The selection process is illustrated in Figure 1.

## Results

### Cardiotoxicity and Chemotherapy

Cardiotoxicity is a condition that occurs when chemotherapy damages the heart. It is a harsh condition that needs immediate medical attention. In order to manage cardiotoxicity, medical professionals employ a combination of strategies, such as reducing the chemotherapy dosage and administering appropriate cardiac medications to patients. Early recognition and treatment of potential heart failure are crucial to ensure positive patient outcomes [11].

The Cardiac Review and Evaluation Committee define cardiotoxicity after these criteria:

Cardiomyopathy with compromised function of the left ventricle:

- Symptoms or signs of heart failure coupled with the presence of third noise, tachycardia, or both;
- Decrease of at least 5% in the ejection fraction with values less than 55% and signs or symptoms present, or a decrease of 10% at values less than 55% in the ejection fraction without signs or symptoms [12,13].

The American Society of Echocardiography identifies cardiotoxicity as a decrease in left ventricular ejection fraction (LVEF) of more than 10% or more than 53% from baseline (mean reference values for two-dimensional echocardiography) [14,15].

**Figure 2:**
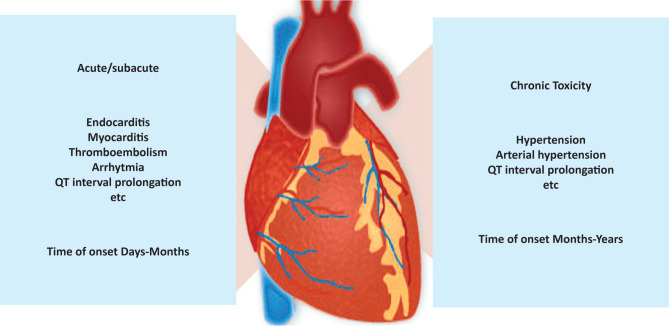
Clinical manifestations of chemotherapy-induced cardiotoxicity.

Chemotherapy is indicated in several phases of antineoplastic treatment as neoadjuvant, adjuvant, or palliative therapy. Therefore, patients may experience a cardiotoxic event early in treatment or up to 40 years after they finish therapy. Therefore, chemotherapy-induced cardiotoxicity is classified into acute or subacute, in which the cardiac damage develops from the onset of treatment and persists for several weeks after its completion. Chronic cardiotoxicity is further divided into two stages: early, within the first year after treatment, and late, which occurs years after therapy has ended (Figure 2) [16,17].

Different drugs and chemotherapeutic agents lead to various degrees of cardiotoxicity, which could be low, moderate, and high risk according to their potency and targeting of cardiomyocytes (Table 1) [18,19].

The cardiovascular expressions of toxic sources include heart failure with ventricle diastolic dysfunction (5-Fluorouracil, Capecitabine, Taxanes, Alkaloids, and Bevacizumab), arterial hypertension (Bevacizumab), arrhythmias (anthracyclines and Taxol), and arterial hypotension (Etoposide, Alemtuzumab, Cantuzumab, Rituximab, and Interleukin 2). The formation of reactive oxygen species (ROS), mitochondrial dysfunction, and changes in calcium and iron homeostasis are some of the putative causes of chemotherapy-induced side effects [18]. These modifications cause cardiac cell death, apoptosis, and angiogenesis inhibition [19,20].

**Table 1:** Antineoplastic drug and risk of cardiotoxicity.

Name of drug	Classification	Risk degree of cardiotoxicity
**Doxorubicin**	Anthracyclines	High
**Cyclophosphamide**	Alkylating agent	High
**Trastuzumab**	Monoclonal antibody	High
**Epratuzumab**	Monoclonal antibody	Moderate
**Docetaxel**	Taxoid	Moderate
**Sunitinib**	Tyrosine kinase inhibitor	Moderate
**Sorafenib**	Tyrosine kinase inhibitor	Moderate
**Masitinib**	Tyrosine kinase inhibitor	Low
**Imatinib**	Tyrosine kinase inhibitor	Low
**Lapatinib**	Tyrosine kinase inhibitor	Low
**Bevacizumab**	Monoclonal antibody	Low

### Types of chemotherapy-induced cardiotoxicity

#### Type I Cardiotoxicity

Type I cardiotoxicity is characterized by sudden, unexpected, and severe changes in cardiac function. Most commonly, it manifests as a decrease in heart rate and pressure as well as an increase in the rate and rhythm of the heartbeat [21]. In severe cases, these changes can result in the weakening or failure of the heart altogether. Other serious effects caused by type I cardiotoxicity include palpitations, dizziness, shortness of breath, and shock. Over time, type I cardiotoxicity can lead to heart failure, characterized by weakening the heart's pumping power. This type of toxicity is related to the damage produced by ROS or free radicals in which the reduction of the quinone group in the B ring of anthracyclines leads to the formation of a semiquinone radical, which is oxidized and generates ROS (e.g., superoxide) [22,23]. This is caused by ROS interacting with the myocardium and producing an imbalance between antioxidant mechanisms and pro-inflammatory substances, which are predisposed to damage by the reduction of glutathione peroxide, which is affected by the use of these drugs [24].

This process is catalyzed by the interaction of Doxorubicin with a ferric iron complex. It causes more ROS to be produced, which helps convert ferrous iron into ferric iron, damaging the endoplasmic reticulum and cell membranes and lowering intracellular calcium and contractility [24-25]. Histamines, TNF-alpha (TNF-α), and interleukin 2 are then released due to inflammatory cytokines. Dilated cardiomyopathy and -adrenoceptor dysfunction are brought on by these cytokines. Topoisomerases have also been linked to the toxicity of anthracyclines in addition to oxidative stress [26]. By establishing a ternary complex with one of the isoenzymes known as Top2a-doxorubicin-DNA, Doxorubicin's anticancer effect is explained. These alterations have a connection to the induction of apoptosis [25].

The most essential and helpful idea is that this group causes early diastolic and late systolic dysfunction as well as dose-dependent (cumulative) myocyte damage [25]. The American National Dogfish Institute defines anthracycline cardiotoxicity as an absolute reduction in LVEF below 50% or a 10% decline in LVEF around the original value, regardless of symptoms or indicators of heart failure. This definition takes into account the cardiotoxic effects outlined. According to the risk classification carried out on each patient, this fact shifted the official indication of clinical-echocardiographic follow-up in a sequential and planned way [26].

#### Type II Cardiotoxicity

Trastuzumab-like type II cardiotoxicity, also known as "effect trastuzumab", is associated with reversible heart damage that permits functional recovery and, if necessary, a resumption of the regimen. Myocytes do not undergo any ultrastructural alterations, allowing this [27,28]. Trastuzumab prevents oral human tum cells that overexpress the HER2 protein from proliferating [29]. It interacts with the extracellular domain of HER2, a transmembrane receptor tyrosine kinase that functions as a proto-oncogene and is connected to the control of cell development. HER2 is an epidermal growth factor. It has a bad prognosis and is overexpressed in 25% of breast cancers [30]. It is connected to neuregulin in the heart, a peptide ligand for HER3 and HER4 that, when combined with HER4, promotes heterodimerization with HER2 and phosphorylation, as well as the activation of several signaling pathways. This boosts the survival and contractile capabilities required for the formation and survival of cardiac myocytes [30] by increasing cell contact and mechanical coupling. Trastuzumab exposure can potentially cause cardiac dysfunction through many molecular processes connected to apoptosis [30,31]. It is crucial to remember that the initial cardio-depressant impact is brief and reversible when the medicine is stopped and that left ventricular ejection fraction (VEGF) recovery takes around a year. Its occurrence varies depending on the risk variables involved; for instance, it ranges from 5 to 30% when administered alone or in combination with anthracyclines. Additionally, this incidence rises with age, a history of cardiovascular illness, and radiation therapy (RT) or chemotherapy (CTX) use in the past, all of which are substantial risk factors for cardiotoxicity. As a result of the finest and strictest risk factor monitoring and the objective to prevent the concurrent use of anthracyclines, the cardiotoxicity in this group has decreased [32].

### Other cytotoxic drugs associated with cardiotoxicity

**Figure 3:**
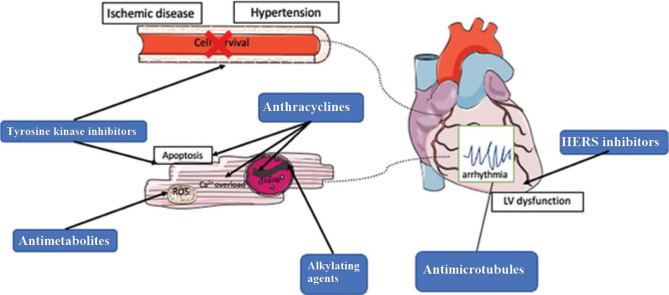
The mechanism of chemotherapy-induced cardiotoxicity.

Cardiotoxicity has been linked to medications including 5-fluorouracil, busulfan, capecitabine, cyclophosphamide, cisplatin, dacarbazine, fludarabine, mechlorethamine, melphalan, mitoxantrone, mitomycin, taxoids like paclitaxel and docetaxel, and monoclonal antibodies like trastuzumab and rituximab [24,33] (Figure 3).

#### Toxoids

After treatment, toxins like paclitaxel and docetaxel have a variety of cardiotoxic consequences. Paclitaxel may cause bradycardia or tachyarrhythmias, atrioventricular and branch blockages, heart ischemia, and hypotension in certain people. This serves as a byproduct of the Purkinje system's direct chronotropic action. The formulation of paclitaxel utilized in clinical settings contains adjuvant chemotherapy, which is also linked to hypersensitivity responses, cardiotoxicity, and nephrotoxicity. Atrial fibrillation, severe coronary artery disease, congestive heart failure, and unstable angina are risk factors for cardiac toxicity [33].

#### Fluorouracil

Fluorouracil (5-FU) is a widely used chemotherapy drug for cancer treatment, but it can cause severe cardiac complications, making it one of the most cardiotoxic drugs. [34]. The exact mechanism that causes 5-FU-induced cardiotoxicity is not fully understood, but it is thought to be linked to the inhibition of thymidylate synthase, an enzyme that plays a vital role in DNA synthesis [34].

#### Cyclophosphamide

Current research on the molecular underpinnings of cyclophosphamide-mediated heart injury may help develop better preventative measures for treating cardiotoxicity. It has been demonstrated that cyclophosphamide treatments suppressed the expression of carnitine palmitoyltransferase-I and heart-type fatty acid-binding proteins in cardiac tissues. Cardiomyopathy results from blocking these pathways, which decreases the amount of adenosine triphosphate produced and causes harmful intermediates from fatty acid oxidation to accumulate. As an early indicator of chemotherapy-induced cardiotoxicity, heart-type fatty acid-binding protein may be employed. Monitoring blood and urine carnitine levels is critical because carnitine shortage can worsen cardiotoxicity. Supplemental carnitine demonstrated positive results in a number of cyclophosphamide-induced toxicities [33,34]. Myocarditis and heart failure (although less common) appear during the first weeks of treatment. A decrease in systolic function also occurs in some patients and, like anthracyclines, increases intracellular concentrations of oxygen ROS [33-35].

#### Cisplatin

The mechanism of cisplatin-induced cardiotoxicity is not fully understood, but it is believed to involve the generation of reactive oxygen species (ROS) and the subsequent damage to cardiac cells. Cisplatin was also found to cause damage to the blood vessels in the heart, leading to heart dysfunction. Additionally, cisplatin causes an alteration in the electrolyte balance which can lead to arrhythmias and cardiac dysfunction. The incidence of cisplatin-induced cardiotoxicity varies depending on the dose and duration of treatment, as well as the patient's age and underlying cardiac conditions. The most common symptoms of cisplatin-induced cardiotoxicity are chest pain, dyspnea, and arrhythmias. In severe cases, cisplatin can cause myocardial infarction and heart failure [8]. Cisplatin can cause severe electrolyte disturbances like hypokalemia, hypocalcemia, and hypomagnesemia, which may trigger abnormal cardiac rhythm [36].

#### Bevacizumab

Thromboembolic events, hypertension, and other cardiovascular problems with wound healing are side effects of Bevacizumab. Higher bevacizumab doses (10–15 mg/kg) are linked to an increased risk of several adverse effects, including thrombosis. According to a meta-analysis, thromboembolism was shown to happen in 11.9% (6.8%-19.9%) of patients with diverse malignant diseases (relative risk [RR], 1.33; 95% confidence interval [CI], 1.13-1.56; pp=.007) and 5 mg/kg per week (RR, 1.31; CI, 1.02-1.68; p=.04). In a separate trial, non-small cell lung cancer (NSCLC) patients receiving first-line treatment experienced fatal pulmonary hemorrhage at both dose levels. Use is prohibited by the current label in NSCLC, including squamous cells. Combination therapy with Bevacizumab increased the frequency of treatment-related deaths (15 patients versus 2 patients; p=.001) compared to chemotherapy alone. Decreased left ventricular ejection fraction was another side effect of long-term Bevacizumab use, while chemotherapy may have also contributed to this [37].

#### Pertuzumab

A humanized antibody called Pertuzumab interacts with trastuzumab by binding to the HER2 receptor at a distinct region. Two different treatment plans with and without Pertuzumab were contrasted by researchers [38]. Compared to the group getting Trastuzumab, Docetaxel, and placebo, the Pertuzumab group had a reduced risk of left ventricular dysfunction (6.6% vs. 8.6%). Trastuzumab was recently studied in the Adjuvant Pertuzumab and Trastuzumab in Early HER2-Positive Breast Cancer Trial (APHINITY TRIAL), which sought to assess its efficacy. Compared to the Pertuzumab/placebo group, the proportion of heart failure was reduced in the Pertuzumab group [38].

#### Interferons alpha

Interferons are cytokines produced from leukocytes (INF-α), fibroblasts (INF-β), and T lymphocytes (INF-γ). INF-α increases the expression of neoplastic antigens on the cell membrane surface to be recognized by the immune cells [21,39]. The use of INF-α can cause arrhythmias ranging from atrial fibrillation to ventricular fibrillation in 20% of patients, and their chronic use can lead to dilated cardiomyopathy [21, 39].

### Risk factors for the development of cardiotoxicity from chemotherapy

Age (children and adults over 65 years old), previous cardiovascular disease, prior radiotherapy (mainly mediastinal), metabolic alterations, and hypersensitivity increase the risk of chemotherapy-induced cardiotoxicity [39]. African Americans are also more susceptible to chemotherapy-induced cardiotoxicity even after adjusting for confounding factors [39]. Likewise, pharmacogenetics can recognize patients with a high risk for chemotherapy-induced cardiotoxicity [40].

### Monitoring and diagnosis of cardiotoxicity

A baseline cardiovascular examination should be conducted to identify the cardiac risk factors of each patient. Before initiating chemotherapy, hypertension and dyslipidemia should be managed [41]. It is recommended to monitor the patient's cardiac health before, during, and after chemotherapy, mainly if anthracyclines were used, to detect any early subclinical changes. However, there are currently no regulations to establish the procedures or frequency for conducting such monitoring [42].

Transthoracic echocardiography is the most commonly used diagnostic technique in oncological clinical practice for measuring cardiotoxicity in patients receiving chemotherapy, as it enables periodic examination of cardiac function and detects any decline in left ventricular ejection fraction (LVEF) [35,42].

On the other hand, the 12-lead electrocardiogram, an early indicator of left ventricular dysfunction in patients receiving extensive anthracycline treatment, exhibits abnormalities in repolarization, decreased voltage of the QRS complex (indicative of cardiomyopathy), and prolongation of the QT interval. Meanwhile, these diagnostic techniques underestimate the severity of heart injury and depend on the operator, and early pharmacological intervention is limited since suggestive alterations caused by cardiotoxicity only surface after serious myocardial dysfunction [42]. In order to properly and promptly identify chemotherapy-induced cardiotoxicity, alternative approaches and procedures have been suggested [32,43].

The European Society of Oncology advises measuring LVEF at the beginning of antineoplastic therapy after half of the total cumulative dose of anthracyclines is administered, before the next dose, and three, six, and twelve months after chemotherapeutic treatment for patients who are older than 60 or have cardiovascular risk factors [35]. Since multiple studies have indicated that changes in LVEF are related to chronic heart failure three years after chemotherapy, antineoplastic therapy should be stopped when the drop associated with an absolute LVEF value of less than 50% is more significant than 10%.

The major disadvantage of this technique is the inability to detect slight differences. Advancements in technology have made it possible for new types of echocardiography to evaluate the myocardial function and detect changes that occur well before the deterioration of LVEF. One of the new echocardiography techniques that have shown promise in detecting early changes in myocardial function is the study of myocardial tissue and the measurement of global longitudinal strain. This technique is helpful in diagnosing subclinical cardiomyopathies and identifying joint bone problems and detecting cardiotoxicity caused by antineoplastic agents [44].

At present, advances in three-dimensional and tissue Doppler echocardiography, myocardial strain imaging, and cardiac magnetic resonance imaging have the potential to detect subclinical changes [31,34, 40]. Endomyocardial biopsy is described in recent publications as the most sensitive and specific method for diagnosing and monitoring cardiotoxicity by anthracyclines since it allows for directly measuring the presence and extent of cardiac fibrosis produced by chemotherapy. Despite its effectiveness, the use of endomyocardial biopsy is limited due to its invasive nature and the need for a blood-based procedure [35].

Assessing LVEF before initiating chemotherapy treatment is a topic of debate regarding the cardiac monitoring of patients. Some authors recommend against performing an initial LVEF assessment in patients who have no cardiovascular risk factors, will receive less than 300 mg/m^2^ of Doxorubicin, are not receiving concurrent trastuzumab treatment, or are females younger than 65 without risk factors. However, some scholars [45] disagree with these recommendations. Due to its relative simplicity, predictability, precision, and accuracy, assessing particular blood biomarkers of cardiac damage has been proposed as an appealing, legitimate, and new technique to identify and monitor cardiotoxicity in patients treated with chemotherapy [45]. Troponin, B-type natriuretic peptide (BNP), and NT-proBNP are the three main serum markers [46]. The "guardian of the genome" serum P53 shields cells from recurring cancer. There are several studies that relate cardiotoxicity and P53 levels [47].

The use of biomarkers, particularly troponin, because of its strong negative predictive value, enables stratification of patients who do not need close monitoring of cardiotoxicity and reduces the use of pointless diagnostic procedures and the expense on the health system and the patients [48].

The same procedures and technique diagnostics utilized in patients with similar symptoms who do not receive chemotherapy may be used to identify additional types of chemotherapy-induced cardiotoxicities, such as ischemia, arrhythmias, and pericardial disease [48]. There is a disagreement on the best course of action for treating these individuals, although prevention techniques for cardiotoxicity have been highlighted. This calls for the need for fresh prospective investigations with sizable patient groups. In addition to developing treatment strategies, researchers must also focus on developing reliable and commercially available screening techniques with biomarkers that can improve risk stratification and cardiotoxicity categorization.

### Treatment and prevention of chemotherapy-induced cardiotoxicity

#### Anthracyclines-induced cardiotoxicity

The independent risk factors for developing cardiotoxicity must be considered to prevent myocardial damage without decreasing the therapeutic efficacy of cancer treatment. Therapeutic schemes can be changed by choosing prolonged infusion application over boluses. Schemes can range from 6–96 hours, and the bolus application's risk of developing cardiotoxicity is 4.13 times greater than prolonged infusion. Administration of drugs in long-time regimens like weekly treatment regimens shows less cardiotoxicity (0.8 vs. 2.9) [2,3, 49]. Utilizing anthracyclines with liposomal coating reduces cardiotoxicity by up to 80% compared to traditional formulations while preventing access into the heart without reducing tumor penetrance [40,49]. The uses of analogs of anthracyclines, such as epirubicin and daunorubicin, have lower cardiotoxicity despite their decrease in therapeutic efficacy [50].

The iron-chelating agent, Dexrazoxane, received FDA approval for preventing anthracycline-induced cardiotoxicity in August 2014. Dexrazoxane acts by binding to iron in the body before entering the cardiac cell and thus decreases the formation of iron-anthracycline complex and decreases free radicals that damage the heart through the peroxidation of lipid membranes with decreased cardiotoxicity in anthracyclines. It is used simultaneously with these drugs or with the first and second doses at baseline when cumulative doses reach 300 mg/m^2^ [50].

### Other chemotherapeutic or radiotherapy agents

#### Digoxin

Several studies have shown that Digoxin is an effective treatment for chemotherapy-induced cardiotoxicities, such as anthracycline cardiotoxicity, due to its ability to suppress oxidative stress and cellular damage [51]. Digoxin is also beneficial when combined with ACE inhibitors to treat Trastuzumab-induced cardiotoxicity [52]. In addition to its cardioprotective effects, Digoxin may have an anti-cancer effect. According to Wang et al., Digoxin can suppress cancer, and its co-treatment with anthracyclines increases their activity [53]. However, the combination of Digoxin and Doxorubicin increases the level of γH2AX, which is associated with DNA changes and induction of apoptosis [53]. On the other hand, a study by Pereira et al. found that the combination of Digoxin and Paclitaxel decreased the anticancer effect [54].

#### Atg7-Based Autophagy Activation

Autophagy is a cellular process that removes damaged organelles and other unwanted material from the cells in the body. Autophagy helps maintain the cell's healthy functioning by removing damaged components and recycling useful materials. This process is also necessary to remove waste materials generated by the cells, such as dead proteins and waste food particles. When autophagy becomes overactive, it can lead to many conditions, including cancer, liver disease, and neurodegenerative disorders [47]. Autophagy, the breakdown of proteins and organelles by autophagic means, is a crucial process for mitophagy, which selectively degrades damaged or dysfunctional mitochondria. In addition to autophagy, mitochondrial dysfunction can also be induced by lysosomes or selective degeneration of other organelles and is considered a core mechanism for mitophagy. Mitophagy is a process in which the autophagic machinery selectively removes damaged or dysfunctional mitochondria. This targeted removal of defective mitochondria helps to reduce cellular damage and improve cellular function and is considered an important mechanism for maintaining cellular homeostasis. Inhibition of this pathway is a common cause of cardiac toxicity and mortality due to introducing a toxic insult to the heart. This effect can be exacerbated by highly toxic chemotherapeutic agents before ATG7 inhibition - especially with Bortezomib, Doxorubicin, and Cyclophosphamide. Other side effects include anemia, hyponatremia, leukopenia, thrombocytopenia, febrile neutropenia, infection, fatigue, rash, gastrointestinal bleeding, and tumor lysis syndrome [54].

#### ATG7 and Doxorubicin

Identifying these specific characteristics of both molecules highlights the potential for the design of improved and less toxic ATG7 and Doxorubicin analogs with reduced cardiomyopathy. However, there are currently no effective treatments to reverse the development of cardiomyopathy in mice despite ongoing research to develop such treatments. The results of this study provide a foundation for identifying new therapeutic strategies in treating cardiotoxicity induced by ATG7 and doxorubicin therapies [48,55].

#### ATG7 activation to decrease cardiotoxicity

There are several studies conducted to evaluate how ATG7 activation can decrease cardiotoxicity. Some studies on experimental animals illustrated that six weeks of Resveratrol with calorie restriction could induce autophagy, which offers some protection against anthracycline-induced cardiotoxicity. Another study on zebrafish found that overexpression of ATG7 at 1-week post-injection prevented and reduced cardiac dysfunction induced by anthracyclines. Additionally, the research observed that daily administration of Spironolactone and Rapamycin restored ATG7 activation, offering cardioprotection to H9c2 cardiac cell lines [48,56].

#### Dexrazoxane to prevent anthracyclines cardiotoxicity

One mechanism behind anthracycline-induced cardiotoxicity is the generation of free radicals, and several studies have implicated iron's role in this toxicity. To counter this, Dexrazoxane offers protection by chelating free radical iron and reducing cardiotoxicity [57]. The FDA approved it in 1995 to prevent cardiotoxicity in patients receiving Cyclophosphamide, Fluorouracil, and Doxorubicin. However, recent studies suggest that taking Resveratrol in patients receiving Doxorubicin significantly reduces free radicals and Doxorubicin cardiotoxicity compared to Dexrazoxane. The mechanism behind Resveratrol's reduction of cardiotoxicity not only involves decreasing free radical generation but also activating ATG7 [58].

#### Monitoring to decrease cardiotoxicity t4

One of the best ways to prevent cardiotoxicity is by monitoring. In March 2017, the American Society of Clinical Oncology (ASCO) published guidelines for controlling and monitoring cardiac dysfunction in adult cancer survivors. The purpose of this guide is to develop recommendations for the diagnosis and monitoring of heart functions in these patients [58].

The guidelines were based on a systematic review of 104 functional clinical studies published between 1996-2016 and focused on clinical methods in the hospital, such as slow IV administration and monitoring, to prevent cardiotoxicity [58].

To identify patients at risk of developing cardiac dysfunction, there should be an assessment of their exposure to anthracyclines and radiotherapy, tyrosine kinase inhibitors, and possibly cardiovascular risk factors (smoking, hypertension, diabetes, dyslipidemia, and obesity) [25,34,58]. Before starting therapy, risks can be prevented or reduced by doing a complete clinical examination of the patient, eliminating any potential cardiovascular risk factors, and avoiding or using cardiotoxic medications as little as possible [25,35,58].

During the course of administering therapy, precautions are taken to reduce danger. Incorporating cardio protectors into treatment (Dexrazoxane) when giving anthracyclines in continuous or liposomal infusions, treating cardiovascular risk factors (smoking, hypertension, diabetes, dyslipidemia, and obesity), and evaluating the fields of therapy and technology to be applied to patients receiving mediastinal radiotherapy are a few examples. Clinical follow-up, imaging tests (cardiac ultrasound, nuclear medicine, and magnetic resonance), assessing blood biomarkers (troponins and natriuretic peptide), and referrals to cardiologists are all used to monitor patients throughout therapy [59]. A detailed medical history, physical exam, and early recognition of cardiotoxic signs and symptoms are required for monitoring individuals at risk for cardiac dysfunction after treatment. Nevertheless, it is suggested that even in asymptomatic patients with left ventricular dysfunction, the causative medication should be discontinued, and appropriate treatment should be initiated if cardiotoxicity is found despite preventive therapy within the predetermined parameters already evaluated by echocardiography or other diagnostic techniques [59].

When symptoms and outright heart failure arise, some studies recommend utilizing medication based on angiotensin-converting enzyme inhibitors and beta-blockers [59]. In symptomatic situations or when rhythm problems are present, appropriate therapy should be designed that incorporates diuretics, aldosterone antagonists, nitrates, and, despite the controversy, Digoxin. The importance of resuming or altering the chemotherapeutic regimen will be considered based on the development and clinical response [59].

#### Medical plant and herbal products that may decrease cardiotoxicity

**Figure 4:**
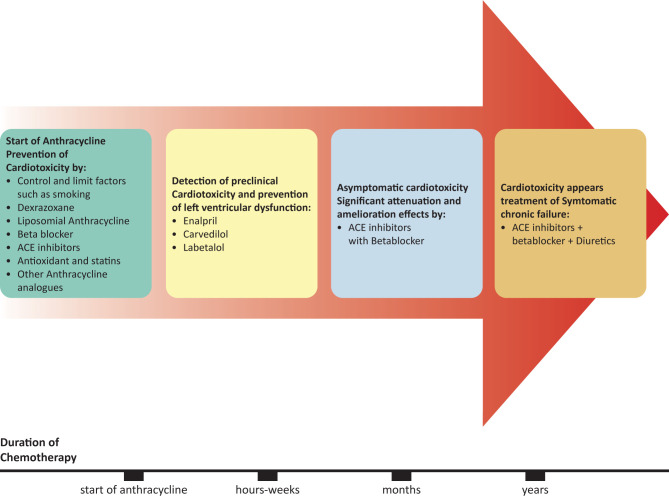
Some strategies to prevent or cure the cardiotoxic effect of Anthracycline induced chronic cardiotoxicity. ACE – Angiotensin Converting enzyme.

The protective effect of grape seed extract has been shown to decrease free radicals and prevent Digoxin toxicity [60]. However, its efficacy as an antioxidant in reducing cardiotoxicity is still in question. Other studies propose the use of Costus pictus extract against anthracyclines toxicity, but when compared with vitamin E, it showed no significant effect [60]. Also, some studies have been conducted on medications such as Febuxostat and herbal plants (e.g., Panax ginseng and pomegranate) that may help to relieve or protect from Doxorubicin cardiotoxicity [4,11,23,61]. Furthermore, several studies recommend using neurohormonal axis inhibitors, such as angiotensin-converting enzyme inhibitors and Carvedilol, which protect from cardiotoxic effects when used during cancer therapy due to their antioxidant effects [4,61], as shown in Figure 4.

## Conclusion

Chemotherapy is often the first line of treatment for many cancers and remains a cornerstone of cancer care. It can be used alone or in combination with other therapeutic techniques. The advances in chemotherapy have significantly increased cancer patients' life expectancy. However, chemotherapy treatment can also have adverse effects due to the treatment's mechanism, dosage, and frequency of administration required to achieve remission. One of the most worrisome side effects of chemotherapy is cardiotoxicity, which can significantly impact a patient's quality of life and long-term prognosis. Recent research has found several drugs and compounds that can lower heart toxicity, including Dexrazoxane, that can reduce chemotherapy-induced cardiotoxicity in high-risk groups. Moreover, Digoxin, ATG7 activators, Resveratrol, and other medical substance or herbal compound have a comparable effect to Dexrazoxane in anthracycline-induced cardiotoxicity. These drugs have demonstrated efficacy in preventing and treating heart toxicity resulting from chemotherapeutic drugs.

## Personal thanks

We would like to extend our sincerest appreciation and gratitude for the valuable collaboration and assistance provided by the Iraqi medical research center.
